# Risk factors for musculoskeletal pain amongst nurses in Estonia: a cross-sectional study

**DOI:** 10.1186/1471-2474-14-334

**Published:** 2013-12-01

**Authors:** Tiina Freimann, David Coggon, Eda Merisalu, Liina Animägi, Mati Pääsuke

**Affiliations:** 1 Tartu University Hospital, Tartu, Estonia; 2 MRC Lifecourse Epidemiology Unit, University of Southampton, Southampton, UK; 3 Department of Public Health, Medical Faculty, University of Tartu, Tartu, Estonia; 4 Tartu Health Care College, Tartu, Estonia; 5 Institute of Exercise Biology and Physiotherapy, University of Tartu, Tartu, Estonia

## Abstract

**Background:**

Routine statistics indicate a high frequency of work-related musculoskeletal disorders in Estonia. We aimed to describe the prevalence of musculoskeletal pain (MSP) amongst Estonian nurses, and to explore associations with personal characteristics and occupational risk factors.

**Methods:**

As a part of an international investigation (the Cultural and Psychosocial Influences on Disability (CUPID) study), a cross-sectional survey was carried out amongst registered nurses at Tartu University Hospital, focusing on pain at six anatomical sites (low back, neck, shoulder, elbow, wrist/hand and knee) lasting for more than a day during the past year and past month. Associations with regional and multi-site (≥2 anatomical sites) pain were analysed by logistic regression.

**Results:**

Analysis was based on 221 female nurses (response rate 57%). The overall prevalence of MSP was 84% in the past year and 69% in the past month. The prevalence of multi-site pain was 60% in the past year and 40% in the past month. Low back, neck and knee were the sites most commonly painful. Pain in the past year tended to be more frequent at older ages, and with higher emotional exhaustion, and at most sites, with poor self-rated health, and reported distress from somatic symptoms. Multi-site pain was also significantly associated with older age and tendency to somatise.

**Conclusions:**

The prevalence of MSP among Estonian nurses is high. Psychological risk factors such as somatising tendency have an important impact. However, none of the risk factors examined seems likely to explain the high frequency of work-related musculoskeletal disorders in Estonia.

## Background

Routinely collected data on occupational diseases in Estonia have indicated a high frequency of work-related musculoskeletal disorders (MSDs) [1]. This makes it important to understand better the causes of MSDs in Estonia, and particularly those which might underlie the high incidence. Nurses are one of the occupational groups which have been found internationally to have relatively high rates of MSDs [2-12], and they might therefore be a useful initial focus for investigation. However, to date there have been no studies of MSDs among nurses in Estonia.

Systematic literature reviews have identified various individual, physical and psychosocial risk factors for common MSDs [13-16], and nursing entails exposure to a number of these factors, including constrained postures, forceful movements, high emotional strain (because of caring for large numbers of patients who may be critically ill), and pressures from staff shortages [4,5,17-19]. A survey in five countries, which included some 43,000 nurses, found that 17–39% planned to leave their job because of its high psychological and physical demands [20,21]. Other psychosocial factors such as time pressures, low job control, lack of support at work, low job satisfaction and insecurity at work, have also been documented as significant risk factors for MSDs amongst nurses [4,22]. Research by Langabelle and colleagues has suggested that burnout, and especially emotional exhaustion, are important determinants of musculoskeletal pain (MSP) among female nurses [23]. However, more evidence is needed about the nature and strength of relationships between MSP and risk factors.

In this study, we aimed to explore the prevalence, localisation and determinants of MSP among nurses in Estonia.

## Methods

As part of the CUPID (Cultural and Psychosocial Influences on Disability) study [24-27], data were collected through a cross-sectional postal survey of nurses at Tartu University Hospital during October 2008 to February 2009. Approval for the study was obtained from the manager of the hospital, and from the Ethics Review Committee on Human Research, University of Tartu. Written informed consent for participation in the study was obtained from all participants.

### Study sample

The study sample comprised 416 individuals, randomly selected from the 869 registered nurses who were employed at Tartu University Hospital at the time of the survey. These nurses were each sent a postal questionnaire, followed by up to two reminders by e-mail, the first after two weeks and the second after one month. Responders were eligible for inclusion if they were aged 20–59 years and had worked in their current job for at least one year.

### Questionnaire

The questionnaire was an Estonian translation of the survey instrument developed for the CUPID study [28], with the addition of supplementary questions on self-rated health and burnout. The accuracy of translation was checked by independent back-translation into English, and amendments were made as necessary. Among other things, the questionnaire covered: demographic characteristics; physical and psychosocial demands of work; somatising tendency; general and mental health; and experience of MSP at six body sites (low back, neck, shoulder, elbow, wrist/hand and knee) lasting for longer than a day during the past year and past month.

Somatising tendency was assessed using questions from the Brief Symptom Inventory [29], and classified according to the number of somatic symptoms (0, 1 or 2+) from a total of seven that had been at least moderately distressing in the past week. Self-rated health was ascertained by the question “What is your overall assessment of your health at present?”, and was classed as good if the participant answered “very good” or “quite good” and poor if the participant answered “average”, “quite poor” or “very poor”. Mood was scored using questions from the relevant domain of the SF-36 questionnaire [30], and was classified to three levels (low, average or high) according to whether the measure was >1 standard deviation below the mean, intermediate, or >1 standard deviation above the mean. An Estonian version of the Maslach Burnout Inventory [31] was used to measure the frequency of psychologically disturbing factors on a scale from 0 to 6, where 0 was not at all, and 6 was disturbing every day. Burnout indicators for emotional exhaustion and depersonalisation were classified to three levels (low, average, or high), again taking cut-points at the mean ± 1standard deviation (personal accomplishment was not analysed because the distribution of scores showed insufficient heterogeneity within the study sample).

Stressful occupational activity was defined separately for each anatomical site, and was deemed to be present if an average working day entailed: lifting weights of ≥25 kg by hand (low back); work with the hands above shoulder height for ≥1 h in total (neck and shoulders); repeated bending and straightening of the elbow for ≥1 h in total (elbow); use of a keyboard or other repetitive movements of the wrist/fingers for ≥4 h in total (wrist/hand); and kneeling or squatting for ≥1 h in total (knees). In analyses of multi-site pain, physical load was considered to be present if the participant reported three or more of these stressful occupational activities.

Questions about time pressure at work were based on the Karasek model [32]. Time pressure was classed as high if the participant reported either a target number of tasks to be finished in a day or working under pressure to complete tasks by a fixed time. Otherwise it was considered low. The questions used to assess MSP were similar to those in the Nordic Questionnaire [33].

### Statistical analysis

Statistical analysis was carried out using the Statistical Package for the Social Sciences (SPSS18.0) and Statistical Software R version 2.12.2. The main outcome measures were pain at each of the six anatomical sites in the past year, and multi-site pain (defined as pain at more than one site) in the past year and past month. Binary logistic regression was used to assess the associations of these outcomes with risk factors, which were summarised by odds ratios (ORs) with 95% confidence intervals (CIs). In each analysis, the referent category was nurses who did not have the outcome under consideration.

## Results

Questionnaires were completed by 237 (57%) of the nurses invited to take part in the study, but 16 respondents were excluded because they had worked in their current job for less than a year or were over 59 years of age. This left a total of 221 nurses who were included in the analysis, and these were employed on 70 wards, 85% as staff nurses and 15% as administrative nurses. All were female and in the age range 23–59 years (mean 38.7 years, standard deviation (SD) 10.2 years). Most (71%) had worked in their job for longer than five years. The mean number of hours worked per week was 40.5 (SD 6.7), and 17% worked more than 40 hours per week.

Among the occupational physical activities that were assessed, the most prevalent was repeated bending and straightening of the elbow (71%) followed by repeated movement of the wrist and fingers (68%) and heavy lifting (38%). Sixty-seven percent of nurses reported time pressures, in the form of a target number of tasks to be completed in a day or working under pressure to complete tasks by a fixed time. The most frequent distressing somatic symptoms were fainting or dizziness (20%), pain in the heart or chest (16%), nausea or upset stomach (16%) and numbness or tingling in parts of the body (17%).

Eighty-four percent of participants reported at least one anatomical site with pain lasting longer than a day in the past year, and 69% MSP in the past month (Table 1). The low back and neck were the sites most often affected by pain, while elbow pain was least frequent. Sixty percent of participants had experienced MSP at ≥2 anatomical sites in the past year, and 40% in the past month.

**Table 1:** Prevalence (%) of musculoskeletal pain in the past year and past month

**Site of pain**	**Past year**	**Past month**
Low back	56.1	39.8
Neck	52.0	38.9
Shoulder	21.3	17.2
Elbow	11.3	6.8
Wrist/hand	27.1	17.2
Knee	32.6	19.5
Number of body sites with pain		
0	16.3	30.8
1	23.5	25.8
2	24.4	19.0
3	19.9	12.2
4	12.2	5.9
5	2.3	0.5
6	1.4	1.4
Missing data for at least one anatomical site	0	4.5

All percentages are calculated on the total sample (n = 221).

Tables 2 and 3 summarise the distributions of the risk factors examined in the study and their associations with pain outcomes. As well as ORs adjusted only for age, mutually adjusted risk estimates are given from regression models that incorporated all of the risk factors in the tables. Although many of the 95% confidence interval included one, pain in the past 12 months tended to be more frequent at older ages (except perhaps at the wrist/hand) and with higher emotional exhaustion. At most sites, it was also associated with worse self-rated health, and reported distress from somatic symptoms (although not always to the point of statistical significance). After adjustment for other risk factors, there were no clear associations with depersonalisation. As regards stressful physical activities, lifting weights ≥25 kg was significantly associated low back pain, and working with the hands above shoulder height with shoulder pain. High time pressure at work was clearly associated only with elbow pain.

**Table 2:** Associations with low back, neck and shoulder pain in past year

**Risk factors**	**Low back pain**			**Neck pain**			**Shoulder pain**		
	**n**	^**a**^**OR (95% CI)**	^**b**^**OR (95%CI)**	**n**	^**a**^**OR (95% CI)**	^**b**^**OR (95%CI)**	**n**	^**a**^**OR (95% CI)**	^**b**^**OR (95%CI)**
**Age (years)**									
23–29	24	1	1	24	1	1	5	1	1
30–39	41	1.2 (0.6-2.5)	1.3 (0.6-2.9)	41	1.2 (0.6-2.5)	1.6 (0.7-3.7)	15	2.2 (0.7–6.5)	3.2 (0.9-11.9)
40–49	29	1.4 (0.7-3.1)	1.8 (0.8-4.6)	26	1.1 (0.5-2.4)	0.8 (0.3-2.0)	14	3.4 (1.1–10.3)	5.1 (1.3-19.6)
50–59	30	3.4 (1.4-8.3)	5.5 (1.8-17.1)	24	1.7 (0.7-3.9)	2.6 (0.9-7.3)	13	4.4 (1.4–13.8)	6.2 (1.4-26.9)
**Self-rated health**									
Good	62	1	1	59	1	1	13	1	1
Poor	62	2.1 (1.2-3.7)	1.1 (0.5-2.3)	56	1.9 (1.1-3.2)	1.2 (0.6-2.4)	34	4.8 (2.3-9.9)	4.4 (1.7-11.4)
**Number of distressing somatic symptoms**									
0	60	1	1	55	1	1	15	1	1
1	31	1.7 (0.9-3.5)	1.5 (0.7-3.4)	27	1.5 (0.8-2.9)	1.4 (0.6-3.0)	15	3.2 (1.4-7.3)	2.0 (0.7-5.2)
≥2	33	3.5 (1.6-7.5)	2.4 (0.9-6.0)	33	3.9 (1.8-8.5)	5.0 (1.8-13.9)	17	4.9 (2.1–11.2)	2.1 (0.7-6.0)
**Mood**									
Good	14	1	1	18	1	1	7	1	1
Intermediate	88	1.6 (0.7-3.4)	1.8 (0.7-4.3)	77	0.8 (0.4-1.7)	0.7 (0.3-1.6)	27	0.6 (0.2-1.7)	0.3 (0.1-0.9)
Poor	22	4.5 (1.5-13.7)	3.4 (0.9-12.5)	20	1.9 (0.7-5.3)	0.8 (0.2-2.8)	13	3.3 (1.0-10.6)	0.7 (0.2-3.2)
**Emotional exhaustion**									
Low	13	1	1	14	1	1	4	1	1
Medium	85	2.1 (0.9-4.7)	2.1 (0.8-5.4)	73	1.2 (0.5-2.6)	0.9 (0.4-2.2)	31	1.9 (0.6-6.1)	2.6 (0.7-10.8)
High	19	2.0 (0.7-5.7)	1.5 (0.4-6.1)	23	3.2 (1.1-9.1)	2.2 (0.5-9.3)	10	3.0 (0.8-11.2)	2.0 (0.3-11.9)
**Depersonalisation**									
Low	13	1	1	6	1	1	3	1	1
Medium	82	0.6 (0.2-1.7)	0.4 (0.1-1.2)	83	2.9 (1.0-8.0)	3.5 (1.1-10.9)	32	1.4 (0.4-5.3)	1.6 (0.3-8-1)
High	21	0.7 (0.2-2.1)	0.4 (0.1-1.6)	23	3.8 (1.2-12.4)	2.6 (0.6-11.3)	11	2.1 (0.5-9.2)	1.7 (0.3-10.9)
**Stressful occupational physical activity**									
No	69	1	1	87	1	1	31	1	1
Yes	54	2.1 (1.2-3.7)	2.2 (1.1-4.3)	27	1.3 (0.7-2.6)	1.1 (0.5-2.4)	15	2.1 (1.0-4.5)	3.0 (1.2-7.3)
**Time pressures at work**									
Low	33	1	1	26	1	1	12	1	1
High	88	1.2 (0.7-2.2)	0.9 (0.5-1.9)	87	1.9 (1.1-3.5)	1.4 (0.7-2.8)	33	1.2 (0.6–2.5)	0.7 (0.3-1.7)

^a^Adjusted for age (age is presented without any adjustment).

^b^Adjusted for all risk factors in the table.

**Table 3:** Associations with elbow, wrist/hand and knee pain in past year

**Risk factors**	**Elbow pain**			**Wrist/hand pain**			**Knee pain**		
	**n**	^**a**^**OR (95% CI)**	^**b**^**OR (95% CI)**	**n**	^**a**^**OR (95% CI)**	^**b**^**OR (95% CI)**	**n**	^**a**^**OR (95% CI)**	^**b**^**OR (95% CI)**
**Age (years)**									
23–29	3	1	1	18	1	1	13	1	1
30–39	6	1.3 (0.3-5.6)	1.4 (0.3-6.5)	19	0.6 (0.3-1.3)	0.6 (0.2-1.4)	19	0.9 (0.4-2.1)	1.1 (0.4-2.7)
40–49	7	2.5 (0.6-10.2)	2.4 (0.5-10.6)	9	0.4 (0.2-1.0)	0.3 (0.1-0.9)	22	2.1 (0.9-4.9)	2.1 (0.8-5.3)
50–59	9	4.6(1.2-18.5)	5.0 (1.1-22.1)	14	1.0 (0.4-2.3)	1.4 (0.5-3.9)	18	2.4 (1.0-5.8)	2.0 (0.7-5.7)
**Self-rated health**									
Good	10	1	1	29	1	1	34	1	1
Poor	15	1.9 (0.8-4.7)	2.1 (0.7-6.2)	31	1.9 (1.0-3.6)	1.5 (0.7-3.2)	38	1.8 (1.0-3.3)	2.0 (1.0-4.4)
**Number of distressing somatic symptoms**									
0	12	1	1	30	1	1	35	1	1
1	7	1.4 (0.5-3.9)	1.3 (0.4-4.2)	15	1.3 (0.6-2.8)	1.2 (0.5-2.7)	20	1.7 (0.8-3.5)	2.0 (0.9-4.4)
≥2	6	1.5 (0.5-4.4)	0.8 (0.2-3.0)	15	1.8 (0.8-3.8)	1.7 (0.6-4.4)	17	1.7 (0.8-3.5)	1.8 (0.7-4.4)
**Mood**									
Good	2	1	1	12	1	1	13	1	1
Intermediate	19	1.8 (0.4-8.5)	1.2 (0.2-6.5)	38	0.5 (0.2-1.2)	0.4 (0.1-1.0)	52	0.7 (0.3-1.6)	0.5 (0.2-1.2)
Poor	4	2.7 (0.4-16.4)	1.3 (0.2-9.9)	10	0.9 (0.3-2.7)	0.6 (0.2-2.2)	7	0.5 (0.2-1.7)	0.3 (0.1-1.0)
**Emotional exhaustion**									
Low	2	1	1	4	1	1	9	1	1
Medium	18	2.3 (0.5-10.7)	1.5 (0.3-7.9)	42	2.9 (0.9-9.0)	3.7 (1.1-12.3)	48	1.3 (0.5-3.1)	1.3 (0.5-3.3)
High	5	2.6 (0.4-14.8)	1.8 (0.2-15.6)	13	5.1 (1.4-18.6)	8.2 (1.6-41.6)	11	1.2 (0.4-3.6)	1.5 (0.4-6.0)
**Depersonalisation**									
Low	2	1	1	5	1	1	7	1	1
Medium	19	1.3 (0.3-6.3)	1.1 (0.2-6.8)	44	1.4 (0.5-4.3)	1.3 (0.4-4.4)	50	0.9 (0.3-2.4)	0.9 (0.3-2.9)
High	4	1.0 (0.2-6.6)	0.7 (0.1-6.5)	8	1.0 (0.3-3.7)	0.5 (0.1-2.3)	12	0.8 (0.2-2.6)	0.6 (0.2-2.7)
**Stressful occupational physical activity**									
No	5	1	1	11	1	1	61		1
Yes	20	2.1 (0.7-6.0)	1.5 (0.5-4.8)	49	1.5 (0.7-3.2)	1.0 (0.4-2.3)	11	0.7 (0.3-1.5)	0.7 (0.3-1.6)
**Time pressures at work**									
Low	2	1	1	15	1	1	26	1	1
High	23	5.6 (1.2-24.7)	5.3 (1.1-26.5)	45	1.3 (0.7-2.6)	1.0 (0.5-2.2)	87	1.1 (0.6-2.2)	1.3 (0.6-2.7)

^a^Adjusted for age (age is presented without any adjustment).

^b^Adjusted for all risk factors in the table.

Table 4 presents findings from regression models for multi-site pain (i.e. pain at ≥2 sites) in the past year, in which risk factors were included if they gave a statistically significant association (p < 0.05) in univariate analyses. Associations are also shown for the same variables in relation to multi-site pain in the past month. Both outcomes were more common at older ages and in association with somatising tendency. An association with poor self-rated health ceased to be significant after adjustment for other risk factors.

**Table 4:** Associations with multi-site pain (≥2 anatomical sites)

**Risk factor**	**Multi-site pain in past year**				**Multi-site pain in past month**	
	**n**	^**a **^**OR (95% CI)**	^**b **^**OR (95% CI)**	**n**	^**a **^**OR (95% CI)**	^**b **^**OR (95% CI)**
**Age groups**						
23–29	25	1	1	14	1	1
30–39	42	1.2 (0.5-2.5)	1.3 (0.6-2.8)	27	1.4 (0.6-3.0)	1.3 (0.6-3.0)
40–49	34	2.0 (0.8-4.4)	1.9 (0.8-4.8)	24	2.3 (1.0-5.2)	2.2 (0.9-5.4)
50–59	32	4.2 (1.7-11.3)	6.3 (2.1-22.7)	23	3.6 (1.5-8.6)	3.7 (1.4-9.9)
**Self-rated health**						
Good	64	1	1	38	1	1
Poor	69	3.2 (1.8-5.9)	1.5 (0.7-3.0)	50	2.6 (1.5-4.6)	1.6 (0.8-3.1)
**Number of distressing somatic symptoms**						
0	62	1	1	36	1	1
1	33	2.1 (1.0-4.3)	1.7 (0.8-3.6)	25	2.5 (1.2-5.0)	2.0 (1.0-4.2)
≥2	38	6.5 (2.7-18.2)	7.3 (2.5-27.2)	27	4.2 (2.0-8.9)	3.1 (1.3-7.0)
**Emotional exhaustion**						
Low	14	1	1	10	1	1
Medium	90	1.9 (0.9-4.3)	1.8 (0.8-4.5)	57	1.4 (0.6-3.4)	1.2 (0.5-2.8)
High	24	3.6 (1.3-11.0)	2.9 (0.9-10.5)	18	2.7 (0.9-7.7)	1.8 (0.6-5.6)
**Physical load**						
Low	72	1	1	47	1	1
High	60	1.8 (1.0-3.2)	1.3 (0.7-2.6)	40	1.7 (0.9-3.0)	1.2 (0.7-2.3)

^a^ Without adjustment.

^b^Adjusted for all risk factors in the table.

## Discussion

This study indicates that, as in many other countries [2-12], regional and multi-site MSP are common amongst Estonian hospital nurses. Moreover, as elsewhere, MSP was associated with tendency to somatise [24,25]. Other significant risk factors for MSP at one or more anatomical sites included poor self-rated health, emotional exhaustion and stressful physical activities at work.

Our method of investigation had the advantage of using relevant subscales from well-recognised and widely used instruments such as the Brief Symptom Inventory [29], Short Form-36 questionnaire [30], and Maslach Burnout Inventory [31]. Moreover, participants were randomly sampled from all nursing personnel at Tartu University Hospital, which is the only university hospital in Estonia.

However, the study also suffered from several important limitations. Some nurses with MSP may leave employment because of their symptoms, leading to under-appreciation of the burden of illness in a cross-sectional survey such as ours. And despite use of reminders, the response to the questionnaire was incomplete (57%). We have no reason to expect that responders would be highly unrepresentative in the relationship between risk factors and pain outcomes, but it is possible that nurses with pain were more inclined to take part in the study. Furthermore, the assessment of exposures was based on self-report. It is possible, for example, that nurses with MSP were more aware of certain physical activities because they exacerbated their pain, and therefore reported them more completely. It could also be that distress caused by MSP made some participants more likely to report emotional exhaustion and poor overall health. However, reverse causation of this type is unlikely to explain the associations which were observed with somatising tendency, which in previous studies has been found to predict the future development and persistence of MSP [24,25]. Another constraint was the modest sample size, which limited the power with which some potential risk factors could be examined.

## Conclusions

Despite limitations, it is safe to conclude that MSP is highly prevalent among Estonian nurses. Indeed, the recorded prevalence of low back and neck pain was approximately twice that which has been reported in Swedish nurses [10]. Furthermore, psychological risk factors such as somatising tendency appear to have an important impact. At the same time, none of the risk factors examined seems likely to explain fully the high frequency of work-related musculoskeletal disorders in Estonia [1]. It may be that the high prevalence is attributable also to culturally determined health beliefs and expectations, a hypothesis which is being investigated in the CUPID study of which this survey formed part. Further study is needed of musculoskeletal pain in nurses, paying particular attention to psychosocial factors in the workplace.

## Competing interests

The authors declare that they have no competing interests.

## Authors’ contributions

TF organised the collection of data, carried out the statistical analysis and drafted the manuscript. DC designed the CUPID study and questionnaire, advised on statistical analysis, and helped to edit the draft manuscript. EM led the Estonian component of the CUPID study, and oversaw data collection, analysis and drafting of the manuscript. LA assisted with the statistical analyses. MP supervised and consulted in the drafting of the manuscript. All authors read and approved the final manuscript.

## Pre-publication history

The pre-publication history for this paper can be accessed here:

http://www.biomedcentral.com/1471-2474/14/334/prepub
